# ﻿*Ooceraeahainingensis* sp. nov.: A new Chinese *Ooceraea* (Hymenoptera, Formicidae, Dorylinae) species with a dealate queen, closely allied to the queenless clonal raider ant *O.biroi*

**DOI:** 10.3897/zookeys.1205.118358

**Published:** 2024-06-20

**Authors:** Qionghua Gao, Jiliang Long, Chengyuan Liu, Haoyu Liu, Hao Ran, Kip D. Lacy, Daniel J. C. Kronauer

**Affiliations:** 1 Guangxi Key Laboratory of Agri-Environmental and Agri-Products Safety/National Demonstration Center for Experimental Plant Science Education, College of Agriculture, Guangxi University, Nanning, 530004, China; 2 Center for Evolutionary and Organismal Biology, Zhejiang University School of Medicine, Hangzhou, 310058, China; 3 Haining Ziwei Senior High School, Haining, 314400, China; 4 State Key Laboratory of Genetic Resources and Evolution, Kunming Institute of Zoology, Chinese Academy of Sciences, Kunming, Yunnan, 650223, China; 5 Laboratory of Social Evolution and Behavior, The Rockefeller University, 1230 York Avenue, New York, NY, 10065, USA; 6 Howard Hughes Medical Institute, The Rockefeller University, 1230 York Avenue, New York, NY, 10065, USA

**Keywords:** Caste, evolution, Formicidae, identification key, systematics, taxonomy

## Abstract

The clonal raider ant, *Ooceraeabiroi*, is a queenless species that reproduces asexually, and these traits make it an attractive model system for laboratory research. However, it is unclear where on the ant phylogeny these traits evolved, partly because few closely related species have been described and studied. Here, we describe a new raider ant species, *Ooceraeahainingensis***sp. nov.**, from Zhejiang, China. This species is closely related to *O.biroi* but can be distinguished by the following features: 1) workers of *O.hainingensis***sp. nov.** have an obvious promesonotal suture and a metanotal groove, whereas these characters are ambiguous in *O.biroi*; and 2) the subpetiolar process of *O.hainingensis* is prominent and anteroventrally directed like a thumb with sublinear posteroventral margin, while in *O.biroi*, it is anteroventrally directed but slightly backward-bent. Molecular phylogenetic analyses confirm that *O.hainingensis* is genetically distinct from *O.biroi*. Importantly, unlike *O.biroi*, *O.hainingensis* has a queen caste with wings and well-developed eyes. This suggests that the loss of the queen caste and transition to asexual reproduction by workers is specific to *O.biroi* and occurred after that species diverged from closely related congeneric species.

## ﻿Introduction

Most ant species live in colonies with two anatomically distinct female castes: queens and workers. Division of reproductive labor between castes has contributed to ants’ ecological success, but some species have lost the ability to produce one of these castes. One example is the clonal raider ant, *Ooceraeabiroi* (Forel, 1907), which has lost the ancestral capacity to produce queens. In this species, colonies are composed entirely of anatomical workers that all reproduce asexually via thelytokous (female-producing) parthenogenesis (development from an egg without fertilization by sperm) (Tsuji and Yamauchi 1995; Ravary and Jaisson 2004; Kronauer et al. 2012; Oxley et al. 2014). These unusual reproductive traits have made *O.biroi* a useful laboratory model species by providing control over genotype in experiments and facilitating genome engineering for functional studies of their social biology (Trible et al. 2017; Hart et al. 2023; Ivasyk et al. 2023; Li et al. 2023). In addition, these traits offer opportunities for comparative study. For example, genomic comparisons between closely related species that retain the capacity to produce queens and species that have lost this capacity might yield insight into the mechanistic basis of caste development.

However, comparative study is currently limited by the lack of knowledge across the genus *Ooceraea* Roger, 1862. Little is known about the biology of *Ooceraea* species other than *O.biroi*, apart from taxonomic species descriptions based on field-collected specimens. This is partly because *Ooceraea* are subterranean and have relatively small colonies, and are therefore rarely encountered. Members of the genus are found throughout tropical and subtropical regions of East Asia and Oceania (Borowiec 2016, 2019; Janicki et al. 2016; Guénard et al. 2017; AntCat 2024; AntWeb 2024), except for *O.biroi*, which has a wide global tropical and subtropical invasive range (Wetterer et al. 2012; Trible et al. 2020). The phylogeny of the 16 described *Ooceraea* species has yet to be resolved (AntCat 2024, AntWeb 2024), and we know little about the reproductive biology of most of these species. Queens have been documented from colony series of five species, including typical dealate queens found in *O.octoantenna* Zhou & Chen, 2020 (see Zhou et al. 2020) and *O.siamensis*Jaitrong et al., 2021, and ergatoid queens found in *O.besucheti* (Brown, 1975), *O.crypta* (Mann, 1921) and *O.quadridentata*Yamada et al., 2018. Queens have not been documented from any other *Ooceraea* species, but this should not necessarily be taken to mean that these species lack queens. Indeed, some species were described only from leaf litter samples [*O.alii* (Bharti & Akbar, 2013)] or single workers [*O.pawa* (Mann, 1919)], rather than from entire colony series. Therefore, it remains unclear where on the *Ooceraea* phylogeny the queen caste was lost.

Here, we expand the knowledge of *Ooceraea* reproductive biology and caste systems by describing workers and a queen of a novel species of this genus from southeastern China. Molecular phylogenetic analysis revealed that this new species is a close relative of *O.biroi*, suggesting that the loss of the queen caste occurred since the common ancestor of these two species.

## ﻿Material and methods

A colony (colony ID: GXU220610) consisting of 17 workers and a dealate queen was collected from the soil of a bamboo forest located at the foot of Yuemiao Mountain, Qianjiang village, Yuanhua Town, Haining County, Jiaxing City, Zhejiang Province, China. The holotype is a pinned worker specimen (individual ID: GXU220610-W-01), preserved in the Insect Collection of Guangxi University (**GXU**), Nanning, Guangxi, China. The paratypes are five workers (individual ID: GXU220610-W-02~06) stored in 75% ethanol at the same institution. These specimens were briefly removed from the ethanol, point mounted, photographed, and then returned to 75% ethanol for preservation. We flash-froze tissue from the queen specimen (individual ID: GXU220610-Q-01) and several worker specimens (individual ID: GXU220610-W-07~17) and stored them at -80 °C before DNA extraction and sequencing.

The *O.biroi* colony (colony ID: GXU230727) used for species comparison was collected from Binqiao Town, Longzhou County, Chongzuo City, Guangxi Province, China. Species identification was based on both morphological characters and *COI* and *COII* genetic information.

We extracted genomic DNA using Qiagen’s QIAmp DNA Micro Kit (California, USA) following the manufacturer’s instructions. PCR amplifications were conducted using the universal primers LCO1490 (5’-GGTCAACAAATCATAAAGATATTGG-3’) and HCO2198 (5’-TAAACTTCAGGGTGACCAAAAAATCA-3’) for *COI* (Folmer et al. 1994), as well as the primers AntLeu (5’- AATATGGCAGATTAGTGCAATGAA-3’) (Kronauer et al. 2012) and Barbara(5’-CCACAAATTTCTGAACATTGACCA-3’) (Simon et al. 1994) for *COII*. The PCR products were then run on agarose gels and Sanger sequenced by Sangon Biotech (Shanghai, China). The resulting sequences were assembled in ContigExpress and aligned with additional, publicly available *Ooceraea* sequences for phylogenetic analysis (see Table 1 for details). The mitochondrial *COI* and *COII* sequences of *Ooceraea* sp. MY08 were obtained from the raw sequence data (BioSample: SAMEA12364593; SRA: ERS9971404) reported in Romiguier et al. (2022). We assembled the complete mitochondrial genome using GetOrganelle (v.1.7.7.0) and annotated it with MITOS. Sequence alignment was performed using the MUSCLE algorithm implemented in MEGA 11 (Tamura et al. 2021). A *COI* + *COII* supermatrix was then constructed using the phylotools package (Zhang et al. 2017) in RStudio. Based on the number of parameters, we selected the “GTR+G+I” model as the best fit for the alignment. Maximum likelihood analysis was then conducted using the GTR+G+I substitution model to estimate the tree topology, and branch support was calculated using the bootstrap method with 1000 replicates.

**Table 1.** Samples used in the phylogenetic analysis.

Samples	Locality	GenBank accession		References
		*COI* 600bp	*COII* 536bp	
Line A *Ooceraeabiroi* isolate C13	Okinawa, Japan	JX157194	JX157205	Kronauer et al. 2012
Line B *Ooceraeabiroi* isolate STC1	Jolly Hill, St. Croix	JX157211	JX157226	Kronauer et al. 2012
Line C *Ooceraeabiroi* isolate C11	Okinawa, Japan	JX157193	JX157204	Kronauer et al. 2012
Line D *Ooceraeabiroi* isolate Cbi48	Tutuila, Am. Samoa	JX157201	JX157212	Kronauer et al. 2012
Line E *Ooceraeabiroi* isolate Cbi25	Uttarakhand, India	JX157196	JX157207	Kronauer et al. 2012
Line F *Ooceraeabiroi* isolate Cbi26	Jammu, India	JX157197	JX157208	Kronauer et al. 2012
Line G *Ooceraea* sp. isolate Cbi6	Nghệ An, Vietnam	JX157195	JX157206	Kronauer et al. 2012
Line H *Ooceraea* sp. isolate Cbi27	Guangdong, China	JX157198	JX157209	Kronauer et al. 2012
Line I *Ooceraeabiroi* isolate BG2	Khulna, Bangladesh	MT086805	MT086822	Trible et al. 2020
Line J *Ooceraeabiroi* isolate BG3	Khulna, Bangladesh	MT086806	MT086823	Trible et al. 2020
Line K *Ooceraeabiroi* isolate BG12	Dhaka, Bangladesh	MT086814	-	Trible et al. 2020
Line L *Ooceraeabiroi* isolate BG13	Lawachara, Bangladesh	MT086815	MT086829	Trible et al. 2020
Line M *Ooceraeabiroi* isolate BG14	Lawachara, Bangladesh	MT086816	MT086830	Trible et al. 2020
***Ooceraeahainingensis* sp. nov.**	Zhejiang, China	PP110965	PP134994	This study
*Ooceraeaaustralis*	Cape York, Australia	JX157199	JX157210	Kronauer et al. 2012
*Ooceraeafragosa*	Sinharaja Forest Reserve, Sri Lanka	MT267599	–	Longino and Branstetter 2021
*Ooceraeaquadridentata*	Dak Lak, Vietnam	LC611729	–	Yamada 2021
*Ooceraea* sp. MY08	Maliau Basin Centre, Malaysia	SAMEA12364593	SAMEA12364593	Romiguier et al. 2022
*Sysciaaugustae* (outgroup)	Honduras	BK012238	BK012238	Allio et al. 2020

We examined the point-mounted specimens using a Nikon 745T stereomicroscope, and took high-quality multi-focused montage images using a Keyence VHX 6000 digital microscope under 200X magnification. We removed artefacts and unnecessary parts of the images and assembled images into figures using Adobe Photoshop CC 2019. The morphological terminology follows Borowiec (2016). We used ImageJ to make morphometric measurements of the following body parts. All measurements are in millimeters.

**HL** Head length: the maximum length of the cranium in full-face view, measured by the straight-line distance from the clypeus’ foremost point, extending to the central point of the cranial posterior margin;

**HW** Head width: the greatest width of the cranium (full-face view, excluding the eyes);

**SL** Scape length: the maximum length of the antennal scape excluding the basal condylar bulb;

**MW** Mesosomal width: the maximum width of the promesonotum in dorsal view;

**ML** Mesosomal or Weber’s length: the maximum diagonal length of the mesosoma in lateral view, measured from the posterodorsal border of the pronotal flange to the posterior basal angle of the metapleuron;

**PL** Petiolar length: maximum length of petiole in lateral view (excluding helcium);

**PH** Petiolar height: maximum height of petiole in lateral view (including subpetiolar process);

**PW** Petiolar width: maximum width of petiole in dorsal view;

**PPL** Postpetiolar length: maximum length of postpetiole in lateral view (excluding helcium);

**PPH** Postpetiolar height: maximum height of postpetiole in lateral view;

**PPW** Postpetiolar width: maximum width of postpetiole in dorsal view;

**CI** Cephalic index: HW/HL × 100;

**SI** Scape index: SL/HW × 100;

**PI1** Petiolar index 1: PL/PH × 100;

**PI2** Petiolar index 2: PW/PL × 100;

**PPI1** Postpetiolar index 1: PPL/PPH × 100;

**PPI2** Postpetiolar index 2: PPW/PPL × 100;

**WI** Waist index: PPW/PW × 100.

## ﻿Results

### ﻿Taxonomy

#### ﻿ Ooceraea hainingensis sp. nov.

Taxon classificationAnimaliaHymenopteraFormicidae

843CBD74-62A6-51D5-9A83-E7B286755B5B

https://zoobank.org/B6AB9D4B-57F8-4758-8903-7DD969206A60

Figs 1 , 2 , 3

##### Etymology.

The species epithet *hainingensis* refers to the type locality.

##### Type material.

***Holotype***: one worker ant; point mounted. Original label: “China, Zhejiang, Haining, Qianjiang village, Yuemiao Mountain, 30.372187°N, 120.810766°E, nesting in the subterranean zone, 10.VI.2022, Haoyu Liu leg.”. ***Paratypes***: five workers from the same colony as the holotype. These type specimens are deposited in the Insect Collection of Guangxi University (GXU), Nanning, Guangxi, China.

##### Description of the workers.

***Measurements and indices***: Holotype: HL 0.53, HW 0.46, SL 0.19, MW 0.32, ML 0.74, PL 0. 0.22, PH 0.36, PW 0.22, PPL 0.25, PPH 0.33, PPW 0.30, CI 87, SI 42, PI1 59, PI2 104, PPI1 75, PPI2 123, WI 135. Paratypes (*N* = 5): HL 0.50–0.56, HW 0.43–0.47, SL 0.18–0.26, MW 0.32, ML 0.66–0.73, PL 0.19–0.22, PH 0.33–0.37, PW 0.22–0.26, PPL 0.22–0.26, PPH 0.30–0.34, PPW 0.27–0.32, CI 82–88, SI 42–57, PI1 52–63, PI2 110–133, PPI1 68–78, PPI2 117–145, WI 117–131.

**Figure 1.**
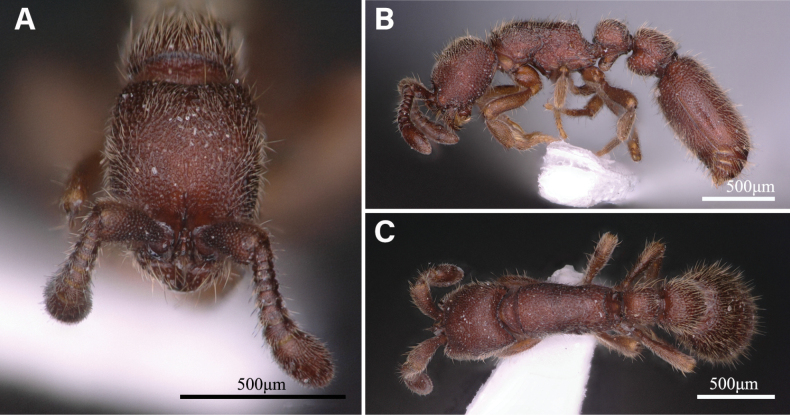
*Ooceraeahainingensis* sp. nov., holotype worker **A** head in full-face view **B** body in lateral view **C** body in dorsal view.

***Head***: In full-face view (Fig. 1A), the cranium subrectangular, distinctly longer than broad; lateral sides weakly/very slightly convex; posterior margin weakly concave medially; posterolateral corners rounded. Mandibles subtriangular, and the masticatory margin lacks distinct denticles. Antennae 9-segmented; scape short and clavate, reaching up to the mid-length of the cranium in full-face view. Antennal sockets fully exposed. Compound eyes and ocelli absent.

**Figure 2.**
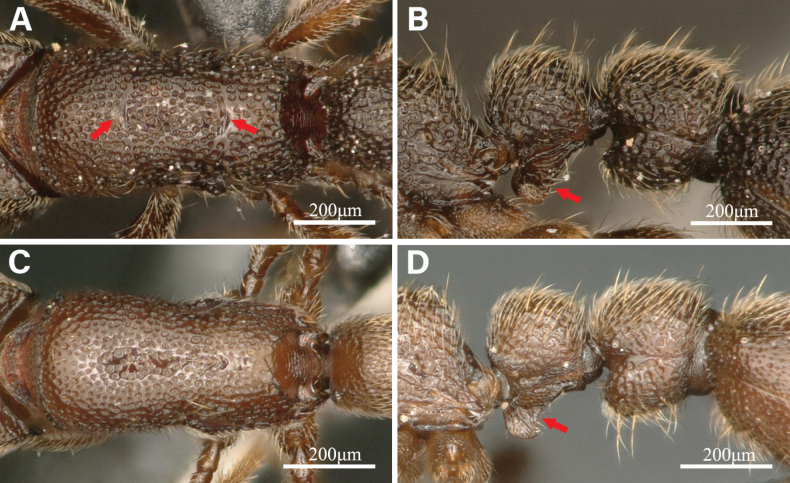
Differences between *Ooceraeahainingensis* sp. nov. and *O.biroi* workers **A***O.hainingensis* mesosoma in dorsal view **B***O.hainingensis* petiole and postpetiole in lateral view **C***O.biroi* mesosoma in dorsal view **D***O.biroi* petiole and postpetiole in lateral view. The red arrows indicate the significant differences between the two species.

***Mesosoma***: Dorsum of mesosoma slightly convex in lateral view (Fig. 1B). Pronotum in dorsal view (Fig. 1C) with anterior margin rounded. Promesonotal suture and metanotal groove present (Fig. 2A). Propodeum in dorsal view with posterior margin concave; propodeal lobe is well formed and roundly shaped (Fig. 2B).

***Metasoma***: Petiole (abdominal segment II) in lateral view (Fig. 1B) much higher than long when including subpetiolar process (PI1, 52–66), with dorsal margin weakly convex. Petiole in dorsal view subrectangular (Fig. 1C), slightly wider than long (PI2, 104–132), with lateral sides weakly convex. Subpetiolar process in lateral view prominent and anteroventrally directed like a thumb, with posteroventral margin sublinear (Fig. 1B, 2B). Postpetiole (abdominal segment III) in lateral view subrectangular, much higher than long (PPI1, 68–78), with dorsal and ventral margin weakly convex. Postpetiole in dorsal view almost trapezoidal, wider posteriorly, broader than long (PPI2, 117–127), and wider than petiole (WI, 117–135), with lateral margins slightly convex. Postpetiolar tergite in lateral view consists of a convex dorsum that is larger than the sternite. The first gastral tergite (abdominal tergite IV) elongated elliptically in dorsal view, with its anterior margin concave and the lateral margin convex.

***Sculpture***: The head, mesosoma, petiole, and postpetiole with dense foveae, with foveae in mesosoma, petiole, and postpetiole slightly larger than in the head in lateral view. Posterior face of propodeum smooth. The first segment of the gaster (abdominal tergite and sternite IV) densely foveolate; with foveae somewhat smaller than those of cranium and mesosoma. Antennal scape and legs micropunctate. Legs roughly shagreened.

***Pilosity***: Body entirely densely covered with decumbent or standing hairs.

***Color***: Body light brown to dark reddish-brown; legs paler.

##### Recognition.

*Ooceraeahainingensis* sp. nov. is readily distinguishable from other described *Ooceraea* species by the following characteristics: 9-segmented antenna; eyes absent in the worker caste; the promesonotum slightly convex; the promesonotal suture and metanotal groove obvious; and the subpetiolar process prominent and anteroventrally directed like a thumb with sublinear posteroventral margin.

*Ooceraeahainingensis* sp. nov. is generally similar to *O.biroi*, but these species differ in the shape of the subpetiolar process, promesonotal suture, and metanotal groove (Fig. 2).

##### Description of the dealate queen.

***Measurement and indices***: Dealate queen (*N* = 1). HL 0.55, HW 0.48, SL 0.25, EL 0.08, MW 0.41, ML 0.81, PL 0.22, PH 0.37, PW 0.22, PPL 0.28, PPH 0.36, PPW 0.25, CI 87, SI 51, PI1 58, PI2 104, PPI1 77, PPI2 92, WI 113.

**Figure 3.**
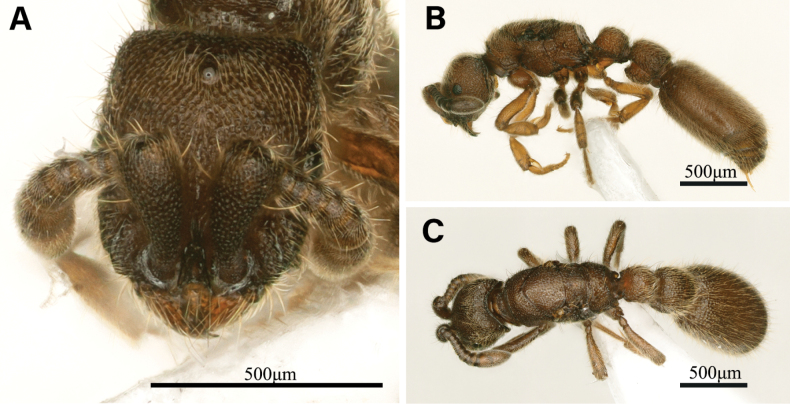
*Ooceraeahainingensis* sp. nov. dealate queen, non-type specimen **A** head in full-face view **B** body in lateral view **C** body in dorsal view.

***Queen description***: Similar to worker in structure, sculpture, coloration and pilosity, but differs from the worker by the following modifications: the body size slightly larger (HW 0.48 in dealate queen, 0.43–0.47 mm in workers; HL 0.55 in dealate queen, 0.50–0.56 mm in workers); compound eyes present approximately at mid-length of the head side; ocelli present and closely approximated (Fig. 3A); mesosoma with unfused flight sclerites.

In lateral view (Fig. 3B), the mesosoma dorsum slightly convex. In dorsal view, the mesoscutum subpentagonal (Fig. 3C), with its lateral sides enclosed by the V-shaped posterior margin of the pronotum; notauli and parapsidal lines absent. The metanotum is narrow. The propodea dorsum with posterior margin concave.

**Male.** Unknown.

##### Habitat.

**Figure 4.**
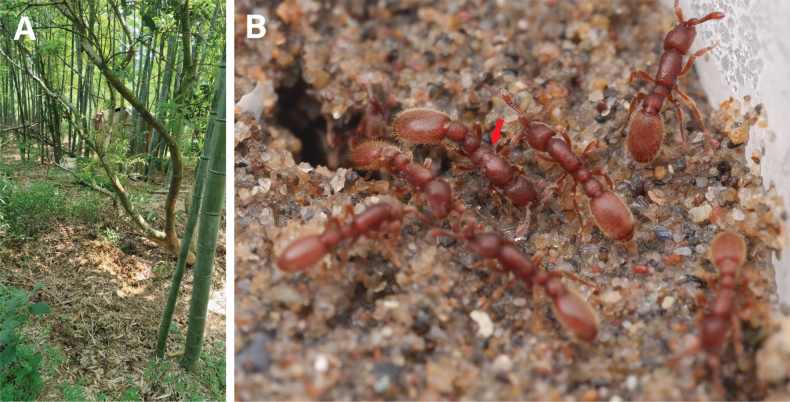
The ecology of *Ooceraeahainingensis* sp. nov. **A** habitat at the type locality of *O.hainingensis* from Haining, Zhejiang Province in China **B** live colony of *O.hainingensis* reared in the lab. The red arrow indicates the dealate queen.

The type specimens are from a colony collected from the Yuemiao Mountain, Haining City of Zhejiang Province in China (30.372187°N, 120.810766°E). The collection site has relatively high canopy cover with low light penetration (Fig. 4A). The temperature at the time of collection was 20 °C. One queen and seventeen worker specimens were collected from the soil in a bamboo forest (Fig. 4B).

##### Distribution.

Only known from the type locality.

### ﻿Phylogenetic analysis

**Figure 5.**
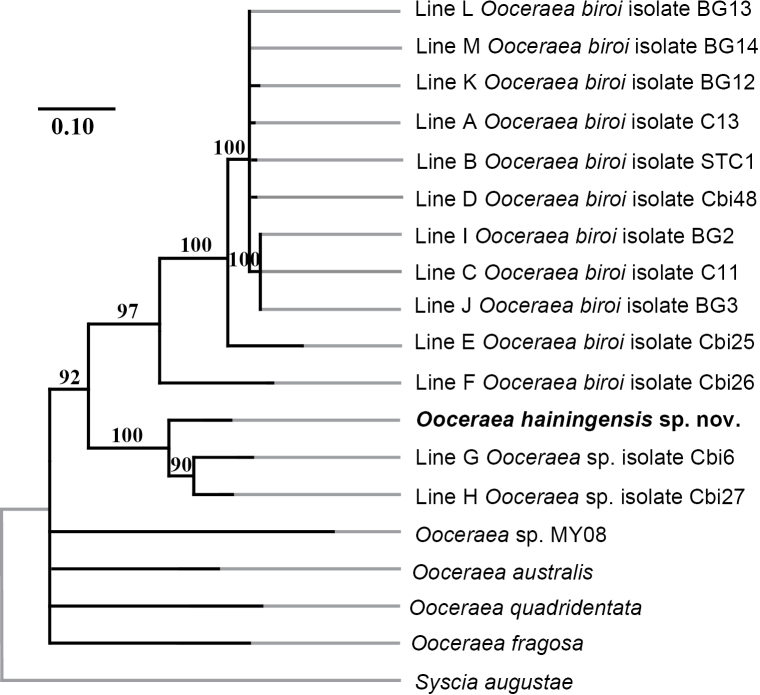
Maximum likelihood (ML) phylogenetic tree of *Ooceraea* species based on *COI* + *COII* sequences, with *Sysciaaugustae* as the outgroup. Numbers above branches indicate bootstrap values, and nodes with bootstrap support < 75 have been collapsed. Phylogenetic branch lengths (black) measured as the number of substitutions per site (see scale bar). The focal species *Ooceraeahainingensis* sp. nov. is highlighted in bold.

The maximum likelihood phylogeny indicates that *O.hainingensis* sp. nov. forms a well-supported clade with Line G and Line H, which represent potentially undescribed *Ooceraea* species that were collected in Nghệ An (Vietnam) and Guangdong (China) (Fig. 5, Table 1). In our analysis, this clade is sister to, and genetically distinct from the *O.biroi* clade, which includes representative sequences of isolates of *O.biroi* from its native range in Bangladesh, more distantly related samples from India, and its invasive range globally. The new species of *O.hainingensis* was further supported by the large percentage of sequence differences, i.e., p-distances, calculated in MEGA (Suppl. material 1).

### ﻿Key to *Ooceraea* of China

**Table:** 

1	Antennae 8-segmented	***O.octoantenna* Zhou et al., 2020**
–	Antennae 9-segmented	**2**
2	Promesonotal suture and metanotal groove ambiguous; subpetiolar process prominent and ventrally directed	***O.biroi* Forel, 1907**
–	Promesonotal suture and metanotal groove obvious; subpetiolar process prominent and anteroventrally directed like a thumb with sublinear posteroventral margin	***O.hainingensis* sp. nov.**

Note: Although the type locality (Shanghai, China) of previously described *Cerapachyssinensis* Wheeler, 1928 (one of the invalid synonyms of *O.biroi*) is very close to the collecting site of *O.hainingensis* sp. nov., they can be easily distinguished by the shape of the subpetiolar process.

## ﻿Discussion

In this study, we describe the worker and queen of *Ooceraeahainingensis* sp. nov., a novel species of *Ooceraea* from southeastern China. Molecular phylogenetic analysis demonstrates that *O.hainingensis* sp. nov. is a close relative of *O.biroi*, an emerging model species that lacks the queen caste and reproduces asexually via thelytokous parthenogenesis. The presence of queens in *O.hainingensis* sp. nov. suggests that the loss of the queen caste occurred in the lineage leading to *O.biroi* after the divergence of these two species. This improves our knowledge of caste evolution within the genus *Ooceraea*, but a comprehensive understanding will require a more complete taxonomic and molecular phylogenetic study.

It remains unclear when asexual reproduction evolved in *Ooceraea*. Because successful lab rearing or genotyping studies have yet to be conducted on any *Ooceraea* species other than *O.biroi*, it is not known whether other *Ooceraea* species reproduce sexually or asexually. Such studies will shed light on whether asexual reproduction is an ancient trait within *Ooceraea* or whether it evolved concurrently with the loss of the queen caste in the lineage leading to *O.biroi*.

We have only scratched the surface of the diversity of reproductive strategies within *Ooceraea*. First, more species likely remain to be described, meaning that continued collecting efforts in the known range of *Ooceraea* may be worthwhile. Indeed, this study marks the second new *Ooceraea* species discovered in China in recent years, including *O.octoantenna* (see Zhou et al. 2020). Counting *O.biroi* means that three *Ooceraea* species can be found in China, and these can be readily distinguished based on the obvious promesonotal suture and metanotal groove and the morphology of the subpetiolar process. Second, more information is needed about the biology of described species. Apart from *O.biroi*, very few collections of *Ooceraea* are recorded in the literature, meaning that we know almost nothing about within-species variation in reproductive strategies. The recent discovery of a queen-like mutant lineage of *O.biroi* highlighted such within-species variation in caste phenotypes, and revealed candidate molecular mechanisms for caste evolution in *Ooceraea* (see Trible et al. 2023). Comparative genomic studies across and within *Ooceraea* species with diverse reproductive biology might help identify mechanisms of caste evolution in ants. Moving forward, the thorough study of the reproductive biology of this genus will be a goldmine for understanding the evolution and mechanistic basis of caste development and thelytokous parthenogenesis.

## Supplementary Material

XML Treatment for Ooceraea hainingensis

## ﻿Additional information

### Author ORCIDs

Qionghua Gao https://orcid.org/0000-0003-2365-1431

Kip D. Lacy https://orcid.org/0000-0002-3149-8927

### Data availability

All of the data that support the findings of this study are available in the main text or Supplementary Information.

## Supplementary materials

Supplementary material 1 Estimates of Evolutionary Divergence between Sequences Data type xlsx Explanation note The number of base differences per site from between sequences are shown. The rate variation among sites was modeled with a gamma distribution (shape parameter = 1.25). This analysis involved 19 nucleotide sequences. Codon positions included were 1^st^+2^nd^+3^rd^+Noncoding. All positions with less than 95% site coverage were eliminated, i.e., fewer than 5% alignment gaps, missing data, and ambiguous bases were allowed at any position (partial deletion option). There were a total of 600 positions in the final dataset. Evolutionary analyses were conducted in MEGA11. https://binary.pensoft.net/file/1074929This dataset is made available under the Open Database License (http://opendatacommons.org/licenses/odbl/1.0/). The Open Database License (ODbL) is a license agreement intended to allow users to freely share, modify, and use this Dataset while maintaining this same freedom for others, provided that the original source and author(s) are credited.Qionghua Gao, Jiliang Long, Chengyuan Liu, Haoyu Liu, Hao Ran, Kip D. Lacy, Daniel J. C. Kronauer
